# Body Composition by CT vs. DXA: Long-Term Prediction of Mortality in the Health Health ABC Cohort

**DOI:** 10.1093/geroni/igaa057.770

**Published:** 2020-12-16

**Authors:** Samaneh Farsijani, Lingshu Xue, Robert Boudreau, Adam Santanasto, Stephen Kritchevsky, Anne Newman

**Affiliations:** 1 University of Pittsburgh, Pittsburgh, Pennsylvania, United States; 2 Wake Forest School of Medicine, Winston-Salem, North Carolina, United States

## Abstract

Background: Early work in the Health ABC cohort found that strength, but not muscle size predicted mortality. Recent literature suggests that body composition by computerized tomography (CT) and magnetic resonance imaging (MRI) predicts adverse health outcomes in diverse populations, but has not been directly compared to dual-energy X-ray absorptiometry (DXA) for predicting mortality. Objective: With long term follow-up, we reexamined body composition and mortality in Health ABC, comparing DXA and CT measures of muscle and fat. Methods: The Health ABC study assessed body composition in 2911 older adults (age 73.6±2.9 years) in 1996-97. Mid-thigh CTs were read for muscle area, inter-muscular, subcutaneous-fat areas and muscle density (HU). DXAs were read for whole body fat mass and appendicular lean mass (ALM). Mortality was assessed every 6-months through 2014 (maximum 17.4 years). Cox proportional hazards models, adjusting for age, sex, race, height, weight, physical activity, smoking and comorbidities were used to assess mortality risk. Results: Strong correlations were observed between mid-thigh muscle and subcutaneous fat areas by CT and leg lean and fat mass by DXA (P<0.05). Lower mortality rates, per SD, were associated with higher CT muscle area (HR-men=0.76 [95%CI: 0.68-0.86]; HR-women=0.84 [0.75-0.94]), muscle density (HR-men=0.86 [0.79-0.93]; HR-women=0.89 [0.81-0.97]) and higher subcutaneous-fat (HR-men=0.90 [0.81-0.99]; HR-women=0.87 [0.77-0.98]), adjusting for covariates. Similarly for DXA, greater ALM (HR-men=0.56 [0.44-0.71]; HR-women=0.77 [0.59-1.01]) and higher total fat mass (HR-men=0.53 [0.40-0.72]; HR-women=0.58 [0.37-0.90]) were associated with lower risk of death. Conclusion: With long term follow-up, both CT and DXA assessments of body composition predicted all-cause mortality risk.

